# Cytopenia among CML Patients on Imatinib in Kenya: Types, Grades, and Time Course

**DOI:** 10.1155/2020/7696204

**Published:** 2020-05-12

**Authors:** Angela McLigeyo, Jamilla Rajab, Mohammed Ezzi, Peter Oyiro, Yatich Bett, Andrew Odhiambo, Matilda Ong'ondi, Sitna Mwanzi, Mercy Gatua, NAOthieno- Abinya

**Affiliations:** ^1^Department of Medicine, Maseno University, Kisumu, Kenya; ^2^Department of Clinical Medicine and Therapeutics, University of Nairobi, Nairobi, Kenya; ^3^Hemato–Oncology Unit, Kenyatta National Hospital, Nairobi, Kenya; ^4^Moi Teaching and Referral Hospital, Eldoret, Kenya; ^5^Aga Khan University Teaching Hospital, Nairobi, Kenya

## Abstract

**Background:**

Imatinib mesylate is the gold standard for the treatment of all phases of Philadelphia-positive chronic myeloid leukemia. Patients on imatinib treatment may develop cytopenia due to drug toxicity. This study aimed to determine the types, grades, and time course of cytopenia in CML patients on imatinib at a Nairobi hospital.

**Methods:**

This was a cross-sectional descriptive study of adult patients aged ≥18 years followed up at the Glivec International Patient Access Program (GIPAP) clinic from 2007 to 2015. Patients who developed cytopenia within 12 months of initiating imatinib were eligible. Clinical and hematologic data were retrieved from the patients' charts and entered into a study proforma. Measures of central tendency such as mean, median, mode, standard deviation, and variance were used for analysis.

**Results:**

Sixty three percent (63.6%) of the 94 patients developed a monocytopenia, with anemia seen in 34%, neutropenia in 27.6%, and thrombocytopenia in 8% of the 94 patients. Anemia plus neutropenia was the most common bicytopenia at 12.7%. Pancytopenia was seen in only 5 of the 94 patients. Most of the cytopenia was grades 2 and 3. Anemia was present at baseline while neutropenia and thrombocytopenia developed within 12 months of imatinib initiation. Anemia resolved during the first 12 months of therapy while neutropenia and thrombocytopenia resolved within 24–36 months of treatment.

**Conclusion:**

Monocytopenia, especially anemia, was the most common type of cytopenia. The cytopenia was predominantly grade 2, developed in majority of the patients within 6 months after imatinib initiation, and had resolved by 24–36 months after imatinib initiation.

## 1. Introduction

Chronic myeloid leukemia (CML) is due to a clonal disorder that causes granulocyte cell line proliferation [1]. It develops following a translocation that occurs reciprocally between two somatic chromosomes, t (9:22) [2]. The fusion protein resulting from this translocation, the BCR-ABL1, is a tyrosine kinase which acts independently of any stimulation [3]. Tyrosine kinase inhibitors (TKI) used in the treatment of CML block this kinase which in turn block signaling pathways involved in proliferation while stimulating apoptosis and cellular adhesion [4, 5]. Patients on imatinib, a TKI, have been reported to develop cytopenia during the course of treatment. Sneed, in their study of 143 CML patients on imatinib, reported that neutropenia and thrombocytopenia ≥grade 3 developed in 64 (45%) and 31 (22%) patients, respectively [6]. A study conducted in Hyderabad among 683 CML patients aged between 21 and 75 years treated with imatinib reported that 46, 25, and 37 patients developed grade 2 anemia, neutropenia, and thrombocytopenia, respectively. Among them, 18 and 13 were reported as bicytopenia and pancytopenia, respectively [7]. The cytopenia was mild with majority of the patients having grade 1 or 2 toxicity [7].

Most of the hematologic toxicities develop but also resolve early following the initiation of imatinib [8] and are potentially reversible with either dose reduction or temporary imatinib discontinuation. Severe hematologic toxicity may occur with higher doses of imatinib used in the setting of imatinib resistance [9]. In the IRIS trial, the development of new onset anemia, thrombocytopenia, and neutropenia after 5 years of follow-up was rare at 4%, 9%, and 17%, respectively, and diminished over time during follow-up. In addition, grade 3 or 4 myelosuppression was infrequent after the initial two years of therapy [10].

In the GIPAP clinic in Nairobi, several patients have been reported to develop cytopenia immediately after imatinib initiation. These patients are managed with either a dose reduction or a treatment interruption. This study aimed to describe the type, grade, and time course of cytopenia among patients treated with imatinib. It will add to the scientific knowledge on types and grades of cytopenia and aid in decision making for these patients.

## 2. Methods

### 2.1. Study Setting

The Max access program provides free imatinib to patients in Kenya at the GIPAP clinics. Cumulatively, the clinic at the Nairobi hospital, Kenya, has enrolled 1200 CML patients. An average of 150 patients attend the clinic bi-weekly. The clinic is centralized and receives patients from the entire country. The age range of patients seen in the clinic is 6 to 75 years. The males that attend the clinic are in similar proportion to the females, and almost 90% are in the chronic phase of CML. Patients who initiate treatment are compliant with treatment with adherence rates of approximately 80% [11].

### 2.2. Study Design and Population

This was a cross-sectional descriptive study of 94 patients. CML patients aged ≥18 years attending GIPAP clinic from 2007 to 2015 and on imatinib 400 mg daily who developed cytopenia ≥grade 2 were enrolled.

### 2.3. Data Collection

Data collection was conducted over a 3-month period. Access to the files was limited to the principal investigator (PI) and a trained study assistant. A coded questionnaire was used as the study instrument to abstract the information from the files. The principal investigator reviewed the data to ensure completeness and accuracy. The patient's names were left out, and instead, each patient was assigned a numerical identifier. Data were extracted from 2007 to 2015.

### 2.4. Data Management and Statistical Analysis

Data were transferred into Microsoft Excel and imported into the statistical analysis software for data management and analysis. Continuous data were presented using means and respective standard deviations (SD). Counts and corresponding percentages were used for categorical variables. Stata package, version 15.1, was used during statistical analysis. Statistical tests were evaluated for significance at the 5% level (*p* < 0.05). Tables, bar charts, pie charts, and line graphs were used to display results.

### 2.5. Variables

Cytopenia was determined from the complete blood count (CBC) report of hemoglobin, neutrophil, and platelet counts. Monocytopenia was defined as an abnormality in one parameter, bicytopenia as an abnormality in two parameters, and pancytopenia as an abnormality in three parameters based. Categorization of severity was based on the National Cancer Institute Common Terminology Criteria for Adverse Events v.3 (NCI CTAE v3) [12].

Time to development of cytopenia after initiation of imatinib was categorized as less than 3 months, 3–6 months, and 6–12 months while duration of cytopenia was determined from annual CBC reports over a 36-month period.

### 2.6. Ethical Considerations

Ethical approval was obtained from the KNH/UON Ethics and Research Committee. Data were stored in a password-protected computer. The study was a minimal risk study since there was no direct patient involvement. For confidentiality, the patients' charts were used only within the confines of the records department of the clinic.

## 3. Results

### 3.1. Type of Cytopenia

Sixty-six patients (63.6 %) among the 94 studied had a monocytopenia. Anemia developed in 32 (34%), neutropenia in 26 (27.6%), and thrombocytopenia in 8 (8%) of the 94 patients. Among patients with bicytopenia, the most common type was anemia plus neutropenia in 12 (12.7%) patients, followed by neutropenia plus thrombocytopenia in 8 (8%) patients and anemia and thrombocytopenia in 3 (3%) patients. Pancytopenia developed in 5 patients among the 94 studied.

### 3.2. Grade of Cytopenia

Grade 2 and 3 cytopenia were equally common at 48.5% and 43.9%, respectively, as shown in Table 1.

### 3.3. Time to Development of Cytopenia

Thirty-seven (40%) patients developed cytopenia within 3 months of initiating imatinib, 33 (34.7%) within 3–6 months, and 24 (25.3%) within 6–12 months.

### 3.4. Duration of Cytopenia

**Figure 1:**
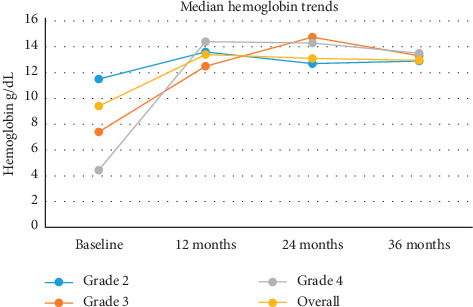
Median hemoglobin trends over a 36-month time period.

**Figure 2:**
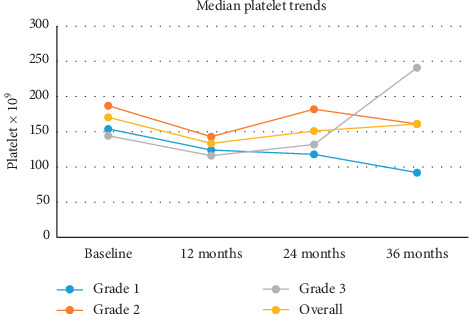
Median platelet values over a 36-month time period.

**Figure 3:**
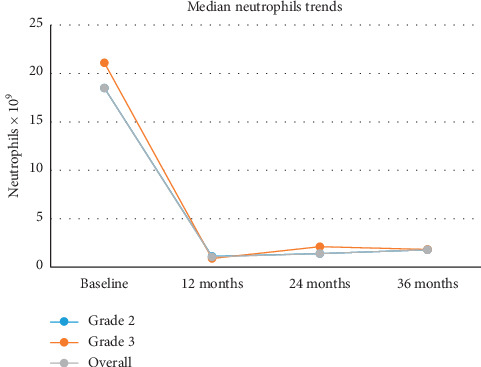
Median neutrophil values over a 36-month time period.

All grades of anemia improved from the reported baseline and resolved within 12 months (Figure 1). Thrombocytopenia improved from 12 months and had resolved by 36 months except in one patient (Figure 2). Neutropenia also began to improve after 12 months for the affected patients and had resolved by 24–36 months (Figure 3).

## 4. Discussion

The study was conducted among 94 patients with cytopenia. More patients developed monocytopenia (71%) than either bicytopenia (24%) or pancytopenia (5%). Anemia was the most common monocytopenia, and anemia plus neutropenia was the most common bicytopenia. Pancytopenia was seen in only 5 of the 94 patients. Grade 2 and 3 cytopenia was common at 48.5% and 43.9%, respectively. Majority of the cytopenia (74%) developed within six months of initiating imatinib and resolved within 12 months for anemia and within 24–36 months for thrombocytopenia and neutropenia. Our data are similar to that from India where anemia developed in 46 (35%), thrombocytopenia in 34 (25%), neutropenia in 24 patients (17%), and bicytopenia in 18 patients. This study included grade 2 to 4 cytopenia in the analysis and was conducted in a low-resource setting similar to ours [7]. Similarly, a different study from India reported that anemia was the most frequent myelotoxicity and occurred in 129 (65%) participants, followed by neutropenia in 57 (28%) and thrombocytopenia in 34 (17%) participants [13]. In another trial, during the first 12 months of treatment, grade 3 and 4 toxicity was noted among the 532 study participants. Neutropenia developed in 33%, thrombocytopenia in 18%, and anemia in 6% of the patients [8]. In contrast, a study conducted in Indonesia reported lower levels of cytopenia with anemia at 20%, thrombocytopenia at 14%, and neutropenia at 4% [14] while a study from Iraqi documented anemia at 14%, and neutropenia and thrombocytopenia at 10% and 4% [15]. Lower proportions of cytopenia reported in some studies may be due to the analysis of patients with grade 3 and 4 cytopenia only. Earlier diagnosis of CML in the developed world may also result in lower levels of hematological toxicity.

Our study reported grade 2 and 3 cytopenia as the most common grade. Similarly, an Indian study reported high proportions of grade 2 cytopenia at 18.5%, 8.8%, and 10.3% for anemia, neutropenia, and thrombocytopenia, respectively, followed by grade 3 at 11.8%, 6.6%, and 7.4% for anemia, neutropenia, and thrombocytopenia, respectively [7]. Druker et al. reported lower levels of severe cytopenia following imatinib therapy with grade 3-4 neutropenia at 14–19%, thrombocytopenia at 8–10%, and anemia at 3-4% [10]. The IRIS trial likewise reported severe cytopenia of grade 3 or 4 to be low with anemia at 3%, neutropenia at 14.3%, and thrombocytopenia at 8% [16]. In contrast, a study from Spain reported grade 3-4 cytopenia as being the predominant grade [17], and Zhou et al. reported grade 3 to 4 neutropenia, anemia, and thrombocytopenia in 21.8%, 17.8%, and 5.9% of patients with CML, respectively [18]. The presence of grade 3-4 toxicity may be due to presentation with advanced CML. Kantarjian et al. reported higher grades of cytopenia in patients presenting with advanced chronic phase-CML, with 35%, 20%, and 7% developing neutropenia, thrombocytopenia, and anemia, respectively, of grades 3 to 4 [19] while a similar study also reported that advanced CML and high imatinib doses of 600 mg were associated with higher proportion of cytopenia [20].

Cytopenia developed within 3 months of imatinib initiation in 40%, 3–6 months in 34.7%, and 6–12 months in 25% of the patients. Myelosuppression may develop at any time during treatment of CML but is common in the initial 2–4 weeks of therapy, especially for advanced disease [21]. Other studies have reported onset of cytopenia between 3 and 5 weeks of imatinib therapy [22].

The development of severe cytopenia rapidly in patients with a myeloid bulge shortly after TKI initiation is a phenomenon that is still under study. The elevation of leucocytes in these patients probably reflects the high disease burden at diagnosis [19]. Once normal blood cell formation resumes, the myelosuppression that has been induced by TKI treatment resolves and the risk of developing myelosuppression later in the course of treatment reduces. In addition, the blood and marrow laboratory results often normalize in the course of follow-up [8]. This was reported in our study where there was recovery of anemia, thrombocytopenia, and neutropenia at the end of the 36-month follow-up. In other studies, grade 3 and grade 4 cytopenia resolved soon after treatment interruption and did not recur with resumption of treatment [23]. The IRIS trial similarly reported that myelosuppression developed early in the treatment course with imatinib therapy but had resolved in the majority of patients after the first 12 months of follow-up [24]. The recovery of the cell counts within 12 months of initiating treatment could reflect bone marrow normalization and regeneration [25]. However, some isolated cases of recurrent myelosuppression requiring extended treatment interruptions have been reported and may be a consequence of advanced disease [17]. Conversely, the cytopenia may recover within days after its development as has been documented prior where the median duration of thrombocytopenia and neutropenia was less than 3 weeks [23]. The duration of cytopenia may also be influenced by duration of CML prior to diagnosis. A study reported that patients with long duration of CML disease prior to diagnosis tended to have more advanced disease. In the event of myelotoxicity in these patients, the duration of cytopenia was more prolonged, especially with higher grades of cytopenia [26].

In conclusion, monocytopenia was common, was of grades 2 and 3, developed early in the course of treatment, and resolved relatively early during follow-up. Clinicians involved in the management of these patients should be alert to diagnose, manage, and monitor these patients.

## Figures and Tables

**Table 1:** Grades of cytopenia among the 66 patients with monocytopenia, NCI CTCAE, v3.

Variables	Grade 2	Grade 3	Grade 4
Anemia (g/dL), *n* (%)	8–10	6.5–8	<6.5
	17 (53)	13 (40.6)	2 (6.4)
Neutropenia (mm^3^), *n* (%)	≥1000–1500	500–1000	<500
	10 (38.5)	14 (53.8)	2 (7.7)
Thrombocytopenia (mm^3^), *n* (%)	50000–75000	25000–50000	<25000
	5 (62.5)	2 (25)	1 (12.5)
Total, *n* (%)	32 (48.5)	29 (43.9)	5 (7.6)

## Data Availability

The datasets used and/or analyzed during the current study are available from the corresponding author on reasonable request.
